# Male Adolescent Substance Use Disorder and Attention-Deficit Hyperactivity Disorder: A Review of the Literature

**DOI:** 10.1155/2013/815096

**Published:** 2012-11-28

**Authors:** Robert Eme

**Affiliations:** Illinois School of Professional Psychology, Argosy University, Schaumburg Campus, 999 Plaza Drive, Schaumburg, IL 60173, USA

## Abstract

Approximately, one-third of male adolescents in treatment for a substance use disorder (SUD) also have an Attention-Deficit Hyperactivity Disorder (ADHD). This strongly suggests that ADHD is a major risk factor for the development of SUD which practitioners must address if they are to provide adequate treatment for adolescents with SUD/ADHD. This paper supports a causal role for ADHD in the development of SUD and examines the developmental mechanisms whereby ADHD increases risk for SUD. These mechanisms include increased risk for conduct disorder, academic failure, deviant peer affiliation, engaging in risk behaviors, and self-medication. Assessment and treatment recommendations for those comorbid for SUD/ADHD are provided.

## 1. Introduction

Substance use, which includes smoking, drinking alcohol, or using other addictive substances, exists on a continuum ranging from no use on one end, to use that does not involve negative consequences, to risky use, to a substance use disorder (SUD) [1]. SUD is characterized by inability to consistently abstain, impairment of behavioral control, craving, and significant problems in behavior and interpersonal relationships [1]. SUDs are a worldwide major public health problem [2]. In the United States, adolescent substance use is arguably, if not incontestably, the number one public health problem for several reasons [3]. Addictive substance use is widespread as almost half of high school students (46.1 percent) are current users (used in the past 30 days) of cigarettes, alcohol, marijuana, or cocaine; one in eight (11.9 percent) meet clinical criteria for a substance use disorder (SUD) [3]. Adolescence is the critical period for the initiation of substance use and its consequences as nine out of 10 Americans who suffer from a SUD started smoking, drinking, or using other drugs before age 18 [3]. The financial and human consequences are staggering. Financially, the cost is estimated as an astonishing $1500 per year for every person in the United States [3]. The human costs include, in addition to heightened risk of addiction, reduced academic performance and educational achievement; criminal involvement; unintended pregnancies; accidents and injuries which make it among the leading causes of death among youth under 21 [3, 4].

Despite being the most important public health problem, only 15.4% of adolescents with SUD receive treatment [5], and this treatment is generally suboptimal [3]. Indeed, there is such a profound disconnect between evidence and practice that “…the vast majority of people in need of addiction treatment do not receive anything that approximates evidence-based care” [1, page 1]. Hence it is not surprising that more than 50% of adults and adolescents who begin treatment drop out or terminate with unsatisfactory progress [1, 4]. For example, in the largest psychosocial treatment study to date of adolescents with SUD (*n* = 600), the Cannabis Youth Treatment Study (CYT), only 25% were in recovery at a 1-year followup, defined as no substance use or dependence problems and living in the community [6, 7]. Similarly, the findings are mixed for the effectiveness of juvenile drug courts [8] and brief interventions with adolescents in acute settings who present with risky alcohol use [9]. An important reason for treatment typically being suboptimal with the resultant bleak outcomes is the failure to treat mental health disorders that co-occur in two-thirds of people seeking treatment for SUDs [1]. A prime example is the failure to identify and properly treat adolescents with SUD who are comorbid for Attention Deficit Hyperactivity Disorder (ADHD) [10]. As the review will document, at least one-third of adolescents in treatment for SUD are comorbid for ADHD and this comorbidity is associated with an earlier onset of SUD, more severe and longer duration of SUD, more difficulty remaining in treatment, and a greater likelihood of relapse after treatment [11–13].

 Despite this high rate of comorbidity, ADHD often goes untreated in adolescents with SUDs [14]. Hence it is critically important that practitioners understand the relationship between SUD and ADHD in adolescents since cooccuring disorders present serious challenges to traditional mental health and substance abuse treatments systems for adolescents [11]. The purpose of this paper is to provide such an understanding for male adolescents as data for females is just emerging [15]. It will do so by first establishing that adolescents in treatment for SUD are frequently comorbid for ADHD. Second, it will discuss the mechanisms that explain this comorbidity. Third, it will provide recommendations for assessment and treatment. Fourth, it should be noted that given the vastness of the literature on SUD and ADHD and given that the goal of the review is to be broadly synthetic, the paper will draw on findings of authoritative critical reviews and meta-analyses as well as individual studies. Lastly, since it is clearly beyond the scope of this paper to address the numerous other risk factors involved in SUD, the reader desiring such a discussion should consult these two current, comprehensive, and authoritative sources [1, 3].

## 2. Prevalence of ADHD in Adolescents in Treatment for SUD

ADHD is the most common neurodevelopmental disorder in juveniles [16] with a prevalence estimate for juveniles in the United States aged 4–17 based upon parental report of ever having been diagnosed with ADHD of 9.5% (13.2% male, 5.6% female) [17]. However, its prevalence among adolescents with SUD is much greater. Aggregate data from studies in the United States yield prevalence rates ranging from 38% to 50% [6, 18]. In the largest sample to date of 7,435 cases in treatment for SUD, most of whom were male and under 18, Conrad and colleagues [19] reported an ADHD prevalence rate of 43% based on DSM-IV criteria assessed at intake. Van-Emmerik-van Oortmerssen and colleagues [2] conducted a meta-analysis of all methodologically sound studies (as of January, 2010) of ADHD prevalence among adolescents in treatment for SUD. The analysis identified 14 studies involving 4054 patients conducted in 6 countries. Studies were only included if ADHD was diagnosed by means of a (semi) structured diagnostic instrument or a systematic DSM-based clinical interview using DSM III or DSM IV criteria. Overall 24.2% (CI. 19.0–30.4%) of the patients met DSM-criteria for comorbid ADHD. Furthermore, since the timing of the evaluation for ADHD is crucial as symptoms of substance intoxication or withdrawal can be mistaken for ADHD symptoms, an analysis was conducted on the 14 studies which explicitly stated that they had assessed for ADHD after a period of at least 4 days abstinence. The findings were the same.

Lastly, as van-Emmerik-van Oortmerssen and colleagues [2], as well as others have noted, because information from family members is typically not part of the assessment procedure for ADHD, the sole reliance upon the self-report of troubled adolescents probably resulted in an underestimate of the true prevalence of ADHD. Thus, in the largest study of juveniles in detention facilities in the United States, Teplin et al. [20, page 1135] remarked that “Attention-deficit hyperactivity disorder (ADHD) is difficult to assess via self-report and is even more challenging to diagnose among delinquents.” For example, Schwaab-Stone et al. [21] in examining the criterion validity of the Diagnostic Interview Schedule for Children Version 2.3. found little more than chance agreement (Kappa correlation of .10) between youth (aged 9–18) report of ADHD symptoms and clinical assessment. Because of this limitation, the most recent authoritative clinical practice guidelines for the diagnosis of ADHD for juveniles (4–18) specify that information should be obtained primarily from reports from “parents or guardians, teachers, and other school and mental health clinicians involved in the child's care” [16, page 1]. Failure to obtain information from parents, teachers (or in the case of adults, a significant other) can be expected to result in substantial under diagnosis not only in juveniles [22, 23], but also adults [24].

The best evidence that sole reliance on self-report results in a substantial underestimate of ADHD comes from the follow-up study into young adulthood (age 21) of 147 males who had previously been diagnosed with ADHD in a childhood [23]. Using a developmentally referenced criteria for diagnosing ADHD in adults resulted in a huge discrepancy when the criteria were assessed by self-report (12%) versus parental report (66%). The correlation between self- and parent-reported levels of ADHD symptoms was just .21. Because parent-reported symptoms were found to have a far greater association with various measure of impairment at age 21 than self-reported symptoms, the study concluded that parent reports provided a more accurate description of current ADHD symptoms and impairments than self-report. Similarly, Young and colleagues [25] conducted a study to determine the most reliable source of information of ADHD symptoms by comparing rating scales scored by 54 male delinquents who were detained in a high risk care home and by their teachers with psychiatric diagnosis of ADHD made by a professional clinical assessment. Sensitivity rates were 33% for delinquent self-report compared to 67% for teacher report. 

In summary, ADHD prevalence rates for adolescents in treatment with an SUD range from 25% to 50%. Moreover, because assessment for ADHD in most studies failed to obtain information from parents and teachers, most studies probably underestimated the true prevalence rate of ADHD. Given this wide range and the high probability of underdiagnosis, Molina [15], one of the foremost experts on the linkage between ADHD and SUD, has estimated that approximately a third of adolescents in treatment for SUD are comorbid for ADHD. The paper will adopt this conservative estimate. 

Among the various conceptualizations that have been suggested to explain this comorbidity, two have the most research support: the secondary substance abuse disorder model and the common factor model [11]. The secondary substance abuse disorder model explains comorbidity by positing that disorders such as ADHD increase risk for the development of SUD. The common factor model posits that high rates of comorbidity are the result of shared risk factors. 

## 3. Secondary Substance Abuse Disorder Model

There is a robust consensus, including two recent meta-analytic reviews, that ADHD is a major risk factor for the development of SUD [12, 13, 26, 27]. For example, in their meta-analytic review, Lee et al. [27] concluded that children with ADHD were at least 1.5 times more likely to develop SUD across diverse forms of substances, including nearly 3 times higher for nicotine dependence.

Four distinct risk mechanisms, discussed in order of importance, are operative in this model. The first is a cascading sequence in which ADHD increases risk for the development of the disruptive behavior disorders of oppositional defiant disorder (ODD) and conduct disorder (CD), thereby increasing the risk for the development of SUD, in part through association with deviant peers. The second is increased risk for school failure caused by ADHD leading to association with deviant peers. The third is that the behavioral and emotional impulsivity of ADHD, independent of comorbid CD, increases risk for SUD. The fourth is the use of legal and illegal substances to self-medicate ADHD symptoms. 

## 4. ADHD as a Risk Factor for CD

It is widely acknowledged that the deviance proneness of children with CD increases risk for the development of SUD [15]. Although there has been a great debate over the best way to integrate the multitude of factors that place a child at risk for developing CD into comprehensive causal models, recently a consensus has emerged for three developmental pathways which have substantial research support [28]. A developmental pathway is the “…orderly behavioral development between more than two problems behaviors with individuals differing in their propensity to progress along the successive problem behavior represented by the pathway during development” [29, page 34]. Note that the conceptualization of a developmental pathway for CD is not deterministic, but refers to a propensity, a probability that CD will develop [29]. One developmental pathway that leads to CD begins with high levels of emotional and behavioral dysregulation that result in problems in the executive control of behavior [28]. This is essentially a description of ADHD predominantly presenting with hyperactive-impulsive symptoms [22, 30–32]. In this model, ADHD behaviors emerge first, followed by ODD behaviors reflecting a pattern of negativistic, defiant, disobedient, and hostile behavior towards authority figures followed by more severe conduct problem behaviors (CD) reflecting a repetitive and persistent pattern of behavior in which the basic rights of others or major age-appropriate societal rules or norms are violated [33–36].

### 4.1. ADHD Increases Risk for ODD

It is widely acknowledged that behavioral impulsivity is a core impairment in ADHD [32]. However, it is only recently that emotional impulsivity/dysregulation has also been recognized as a core ADHD impairment [31, 37]. These twin impairments commonly result in symptoms such as irritability, impatience, anger, low frustration threshold, and reactive aggression [31, 38] which greatly increase the risk for coercive, oppositional interchanges [22, 39–41]. Indeed, it is estimated that a typical child with ADHD has an astonishing half a million of these negative interchanges each year [42]. These negative interchanges result in a formal diagnosis of ODD for 52% of juveniles who have been diagnosed with ADHD in combined community and clinical settings [32]. Furthermore, Barkley [31] has observed that having ADHD virtually creates a borderline case of ODD in children. 

Perhaps the most persuasive evidence that, because ADHD is a disorder of impaired executive functioning, that is, impaired self-control/self-regulation [31], it increases the risk for ODD and thereby increases the risk for CD (which will be subsequently discussed), comes from the most recent findings of the landmark Dunedin Multidisciplinary Health and Development study [43]. This longitudinal study, which followed a complete birth cohort of 1,037 children from birth to age 32, found that self-control assessed during the first decade of life predicted increased risk of criminal offending at age 32 after controlling for IQ and social class origins. When the sample was segmented into the highest and lowest fifths on self-control, the lowest fifth had much higher crime conviction rates than the highest fifth: 43% versus 13%. Since the measures used to assess self-control were essentially measures of the core features of the behavioral and emotional impulsivity that characterize ADHD (hyperactivity, impulsivity, inattention, lack of persistence, impulsive aggression, and low frustration tolerance) in effect what the study found was that children with many symptoms of ADHD (although they were not formally diagnosed as such in the study) were at high risk for criminality compared to those with good self-control. Thus impressive support has been added to the consensus that “self-control theory is one of the most thoroughly researched and cited theories of deviance, delinquency, and crime” [44, page 245] and that impulsivity is the most crucial variable that predicts antisocial behavior [45].

### 4.2. ADHD/ODD Increases Risk for CD

The role of ODD as a developmental precursor to CD has been well documented [46–48]. Moreover, it is now understood that far from being simply a benign, milder form of CD, ODD plays a key role in the development of CD and is one of the strongest predictors of the onset of CD and of the course of CD symptoms over time [35]. And, although the majority of children with ODD do not go on to develop CD [35], if childhood onset CD develops, it is almost always preceded developmentally by ODD [49]. This development is likely to occur when social contexts increase rather than decrease the antisocial propensity of ADHD/ODD [40, 50–52]. Among the various social contexts which increase the antisocial propensity, association with deviant peers is perhaps the most important, as deviant peer affiliation is one of the best predictors of adolescent substance use [53, 54]. Thus, there is substantial evidence from numerous studies that antisocial youth selectively affiliate with deviant peers, and this association in turn increases the antisocial propensity by providing new opportunities to engage in behaviors such as the use and abuse of various substances [55].

In addition, there is emerging evidence that there are subdimensions of ODD symptomatology that are not equally associated with the risk of developing CD [37]. The symptoms that index a negative affect dimension predict internalizing problems whereas oppositional symptoms such as *often argues with adults, often actively defies or refuses to comply with adult's requests or rules*, which index a “headstrong” dimension, predict CD [37]. The “headstrong” dimension of ODD has been found to be associated with ADHD [56] and thus provides even more support for a developmental pathway to CD which begins with ADHD.

As a consequence of this pathway, 22% of those with ADHD develop CD based upon combining data from community and clinical samples [32]. Furthermore, in clinic studies of child onset CD, the vast majority of males with CD have been found to be comorbid for ADHD [15, 47, 57–60]. As Molina [15, page 193] concluded in her literature review of the relationship between ADHD and delinquency, “the difficulty of identifying children with conduct problems without symptoms of ADHD, or traits akin to ADHD such as impulsivity and distractibility, has strengthened the argument that inattention, hyperactivity, and particularly impulsivity, contribute to the development of serious conduct problems for a substantial portion of delinquent youth.” 

### 4.3. ADHD Increases Risk for School Failure and Association with Deviant Peers

School failure has long been recognized as a risk factor for SUD [15]. Academic functioning is a domain of tremendous difficulty for youth with ADHD such that approximately 30% repeat a grade, 30–40% may be placed in special education, 45% may be suspended from school, and 10–35% may drop out [22]. 

 ADHD increases risk for academic failure in the following ways. First, the severe impairments in selective attention, working memory, and sustaining attention that characterize ADHD adversely affect academic performance [22]. Secondly, the development of ODD in 52% of youth with ADHD further exacerbates these severe academic difficulties [22]. Thirdly, the combination of ADHD/CD plus academic failure leads to a loss of self-esteem, frustration and peer rejection. Consequently they are less likely to be socialized into academic pursuits that direct them away from negative social influences and more likely to associate with deviant peers who in turn are more likely to be substance-using and substance-tolerant [15, 54, 61].

### 4.4. SUD as Self-Medication for ADHD

Although the increased risk that ADHD poses for the development of CD is the most important pathway that leads SUD, it is clear that ADHD can increase risk for SUD independent of increasing risk for CD [15, 62–65] through the mechanism of self-medication. There is significant evidence that a subgroup of adolescents with ADHD may be using licit and illicit substances for the purpose of self-medication (though not necessarily consciously so) which can be defined as the use of substances for reasons other than their euphoric properties, such as ameliorating ADHD symptoms of inattention [12, 15, 66]. 

The biological basis of this self-medication can be explained by the linkage of ADHD and substances to the neurotransmitter dopamine. There is consistent research support that links ADHD to a deficiency in dopamine [67, 68]. Also, all substances of abuse (both legal and illegal) exert their rewarding effects by increasing dopamine [69–71], although the specific pathways used by the different drugs to increase dopamine vary among the drug classes [71]. Thus there is solid scientific evidence that drugs of abuse can function as a form of self-medication because, like stimulant medication, they acutely but temporarily raise the concentration of dopamine in the brain and hence can temporarily improve ADHD symptoms [72]. Unlike stimulant medication, however, drugs of abuse can also cause euphoric effects by triggering a massive dopamine surge followed by rapid clearance [73]. 

Lastly, perhaps the most impressive evidence that some SUD has it origins as self-medication comes from research on nicotine (tobacco, cigarette smoking) and ADHD. Since cigarette smoking has been shown more to be more consistently associated with childhood ADHD than any other substance and less likely than other substances of abuse to have this association explained by CD or antisocial comorbidity, it suggests that the ADHD-tobacco association may be explained by a vulnerability specific to nicotine rather than access to drugs of abuse in deviant peer groups [15]. The possibility of a specific vulnerability is strongly supported by fact that research is being conducted using nicotinic agents to treat ADHD [13]. Thus, while initial exposure in adolescence to cigarette smoking is most likely to be affected by numerous factors such as parental smoking and deviant peer association, the progression to habitual use and addiction may be due to the positively reinforcing, self-medicating effects of nicotine [15]. Support for this theory comes from studies demonstrating that nicotine administration enhances attention in smoking and nonsmoking adults with ADHD to a degree comparable to methylphenidate [74]. Moreover, evidence that nicotine can prime the brain, making it more susceptible to developing addiction to illegal substances [1], provides support for the theory that cigarette use often provides a “gateway” to other drugs such as marijuana [15, 18] which may also serve a self-medicating function. For example, the following interview excerpt (from the author's private practice) of a male adolescent who smoked marijuana several times a day for years and was known at school as the “Weed God” illustrates this possibility.

*Interviewer: What is it like when you go to school high?*

*Client: I can sit there and it helps me concentrate. It's like medicine.*

*Interviewer: How does it help you concentrate?*

*Client: I do not know, it just does. I can concentrate.*

*Interviewer:  Do you have concentration problems otherwise?*

*Client: Yeah.*

*Interviewer: Can you explain those to me?*

*Client: I do not know, like I get distracted easily but when on weed I focus more.*

*Interviewer: Will you tell me more about being distracted?*

*Client: I do not know. I do not, like…pay attention. I'm fidgety. I cannot, like, when I do not smoke weed all I do is like shake my leg or something. I cannot calm down. I use it more—I think I use it more as a medicine than I do to have fun.*

## 5. Common Factor Model

The common factor model posits that high rates of comorbidity are the result of shared risk factors [11]. With regard to ADHD and SUD, both of which can be aptly characterized as disorders of impaired control [75], two of the most important shared risk factors which impair control are the neurobehavioral traits of impulsivity and sensation-seeking [75–78]. These traits characterize ADHD [22, 31, 67, 79, 80], markedly increase risk for SUD, [81], and, as will subsequently be discussed, are mediated by neuronal brain circuits that are similarly impaired in ADHD and SUD [76, 80]. 

## 6. Impaired Control/Impulsivity

Impulsivity refers to the difficulty in withholding a prepotent response (i.e., impaired response inhibition which might be labeled “cannot stop”) [77]. With regard to ADHD, there is a substantial literature which links impaired frontostriatal connectivity to impaired response inhibition [82]. A similar impairment has been found in SUD [83], but until very recently there has been the question of whether the impairment was due to the SUD or predated drug-taking, rendering individuals vulnerable for the development of an SUD. The study by Ersche and colleagues [83] provides strong support for this impairment predating drug-taking. They indentified abnormalities in frontostriatal connectivity not only in addicted individuals but also in their nonaddicted siblings as compared to control group of healthy nonaddicted unrelated individuals. Despite having the same vulnerability, it is not clear why one sibling became addicted and the other did not. Volkow and Baler [75] suggested the possibility of different environmental exposures, different resilience factors, or additional neurobiological vulnerabilities.

## 7. Impaired Control/Sensation-Seeking

Sensation-seeking can be defined as the seeking of varied, novel, complex, and intense sensations and experiences and the willingness to take physical, social, legal, and financial risks for the sake of such experience [84]. As with ADHD/CD, this trait increases risk for SUD primarily by increasing risk for affiliation with deviant peers in that juveniles high in sensation-seeking will tend to seek out peers who can provide opportunities for novel, nonnormative stimulation such as substance use [53]. 

 Sensation-seeking is thought to result from an impairment in the dopamine reward system that characterizes ADHD and SUD [10, 67, 68, 70, 77, 85]. In ADHD, this deficiency, in turn, results in a blunted response to typical rewards, an accentuated response to novelty [67], and thus creates a “craving” for more intense, exciting experiences. For example, as Hallowell and Ratey noted [79, page 25], “many of us with ADHD crave high-stimulus situations. In my case, I love casinos and horse races. Obviously a craving for high stimulation can get a person into trouble.” In SUD, drugs can be used to compensate for this deficiency (reward deficiency hypothesis) [70, 77].

## 8. Recommendations for Assessment and Treatment of Adolescents with SUD/ADHD

Unfortunately adolescents with comorbid disorders often fail to receive effective treatment, if any at all [11]. If this dismal record is to be rectified with regard to adolescents comorbid for SUD/ADHD, the essential first step of treatment is obviously premised on the identification of such individuals. 

## 9. Assessment of ADHD

 Assessment for ADHD should be integrated into a comprehensive psychiatric, addictive, social, cognitive, educational, and family evaluation [86]. Failure to do so, with sole reliance on prior clinical records for diagnosis, can be expected to result in a vast underestimate of ADHD. For example, McAweeney and colleagues [87] found a huge difference when the prevalence of ADHD was solely determined by a prior diagnosis in the clinical record (3%) and after a thorough clinical assessment (44%).This assessment should be conducted in accordance with the following guidelines. 

First, all of the adolescent's existing documentation should be examined for a prior diagnosis of ADHD in childhood. If it does contain a prior diagnosis, a referral for a more comprehensive confirmatory evaluation is warranted since most children diagnosed with ADHD continue to have the disorder into adolescence [22, 88]. 

 Second, since acute intoxication or withdrawal symptoms may mimic ADHD symptoms, such as impulsive behaviors, concentration difficulties or restlessness [89], at least 1 month of abstinence is essential before an evaluation can accurately and reliably assess for ADHD symptoms based upon adolescent self-report or observation [86]. However, much more importantly, since adolescents with ADHD provide notoriously inaccurate self-reports of symptoms and related impairments [88, 90], it is essential that information also be obtained from the adolescent's parent or caretaker and ideally also a core academic teacher [16, 88].

 Third, if the prior screening suggests the possibility of ADHD, the adolescent should be referred to an expert for a confirmatory evaluation.

## 10. Treatment of SUD/ADHD

 If a diagnosis of ADHD is made, it is essential to treat this disorder along with the SUD in an simultaneous, integrated approach, with SUD treated first [10, 11, 86]. The key question is how. Although there is a substantial literature on evidence-based treatments for either SUD [1, 3, 4, 91] or ADHD [16, 42, 92], there are no evidence based guidelines for how these treatments should be integrated for both disorders [11, 18]. Furthermore, clinicians may be reluctant to incorporate stimulants (the first line of treatment for ADHD in juveniles, APA [93]) into treatment for ADHD in adolescents with SUD because of concerns regarding safety and misuse [14]. This issue and the issue of treatment efficacy follows.

## 11. Concerns Regarding Safety and Misuse 

First, some concerns can be laid to rest. The concern of whether the widespread use of stimulant medications to treat children with ADHD increases risk for SUD later in life has been decisively answered. A substantial body of literature has found that stimulant treatment begun in childhood or early adolescence does not increase susceptibility to SUD [15, 18, 86, 94]. These findings may be explained in part by evidence that stimulants act somewhat differently with respect to abuse potential in patients with ADHD [73]. However, the findings are mixed with regard to whether or not stimulant treatment reduces liability to developing SUD [15]. For example in the landmark Multimodal Treatment Study of children with ADHD (MTA), although 14 months of state-of-art treatment combining an optimal medication regimen with intensive behavior therapy was highly successful in that 68% achieved normal functioning at the end of treatment [95], 36 months after treatment began, this group (at ages 11–13) had higher rates of substance use (17.4%), mostly tobacco and alcohol, than an local normative comparison group (7.8%) [96]. (The MTA study is the largest and most comprehensive study of both stimulant medication and behavior therapy of children with ADHD that has ever been conducted. It was a randomized clinical trial that lasted for 14 months of four treatment strategies for 576 children aged 7–9 with ADHD: medication management, behavioral treatment, a combination of these two, and an active control condition based on usual treatment available in the community.) Additional concerns regarding adolescents using drugs and alcohol while taking stimulant medication may also not be warranted, as there does not appear to be an increase in severe drug reactions between stimulants and drugs or alcohol [14, 97]. Also, Winhusen and colleagues [98] found that adolescents with SUD/ADHD who were treated with stimulant medication (osmotic-released methylphenidate) OROS-MPH compared to placebo controls did not misuse the medication, nor experience differences in cravings for their medication or other substances 

Second, legitimate concerns regarding safety and misuse should be addressed by a careful monitoring for signs of possible abuse or diversion such as missed appointments, repeated requests for higher doses and a pattern of “lost” prescriptions [73]. Problems of potential abuse of stimulant medication can be reduced in two ways. First, a prodrug or long-acting formulations can be used, as they are less easily manipulated than immediate release formulations to enable abuse by intranasal or intravenous methods to produce an euphoric effect [73]. (A prodrug is a medication that is initially inactive when first administered and is converted into an active form in the body by the normal metabolic processes involved in digestion.) Second, recently developed novel delivery systems such as the crush resistant shell of Concerta (methylphenidate) or a methylphenidate skin patch can be used [99]. 

## 12. Treatment Efficacy

There have been only a few studies to date which have combined medication and psychological treatment (three open, *n* = 42 subjects and five controlled, *n* = 557 subjects) [18, 94] with the most important comprehensive study being the National Institute of Health multisite study of outpatient treatment of 303 adolescents (aged 13–18 years) comorbid for SUD/ADHD [14, 98, 100]. Almost all subjects met criteria for cannabis abuse or dependence (96%), following by lower rates for alcohol abuse or dependence (56%), and much lower rates (<10%) for other substances such as cocaine and amphetamines. In this 16-week randomized, placebo-controlled study, the adolescents received two treatments: OROS-MPH plus weekly cognitive behavior therapy (CBT) versus placebo plus weekly cognitive behavioral therapy. The primary substance outcome measure was the number of days of past-28-day of adolescent reported nontobacco drug/alcohol use, assessed using a timeline followback procedure (TLFB). (This procedure involved asking adolescents to retrospectively estimate their nontobacco drug/alcohol use at 28-day intervals over the 16-week treatment time period.) OROS-MPH plus cognitive behavior therapy did not show greater efficacy than placebo plus CBT for ADHD or reduction in substance use. Furthermore, despite a modest decline in non-tobacco substance use from the beginning to the end of study, most adolescents in both groups were nonabstinent in the final 28 days of treatment interval, averaging 8–10 days of nontobacco drug/alcohol use. A subsequent secondary analysis was conducted to investigate potential predictors of treatment outcomes with the major outcome measure being whether or not the subject was an “SUD responder” defined as achieving a 50% reduction in substance use days from baseline to week 16 based on self-report on the TLFB. The major finding was an interaction effect which showed that OROS-MPH improved SUD outcomes in adolescents with comorbid CD compared to placebo with 42% of the OROS-MPH group being classified as responders versus 18% of the placebo group. The authors concluded that while this finding is only preliminary, it does suggest that OROS-MPH “may be a useful and effective approach when combined with cognitive behavioral therapy for reducing drug and alcohol abuse in these adolescents” [100, page 5]. They also speculated that more intensive treatments involving day treatment or residential care may produce better results. In summary, integrated treatment of SUD/ADHD having just begun, much work needs to be done to develop and evaluate efficacious strategies [18, 94].

## 13. Conclusion

 The literature provides robust support for the extensive agreement that ADHD is a major risk factor for the development of SUD. This risk results in approximately one-third of male adolescents in treatment for SUD being comorbid for ADHD. The developmental mechanisms whereby ADHD increases risk for SUD include increasing the risk for conduct disorder, academic failure, deviant peer affiliation, engaging in risky behaviors, and self-medication. This widespread comorbidity indicates that assessment for ADHD should be integrated into a comprehensive psychiatric, addictive, social, cognitive, educational, and family evaluation which should be provided for all males with SUD. If a diagnosis of ADHD is made, it is essential to treat this disorder along with the SUD in an simultaneous, integrated approach. Integrated treatment of SUD/ADHD has just begun, and much work needs to be done to develop and evaluate efficacious strategies.
